# A Micro-Level Compensation-Based Cost Model for Resource Allocation in a Fog Environment

**DOI:** 10.3390/s19132954

**Published:** 2019-07-04

**Authors:** Sudheer Kumar Battula, Saurabh Garg, Ranesh Kumar Naha, Parimala Thulasiraman, Ruppa Thulasiram

**Affiliations:** 1 Discipline of ICT, School of Technology, Environment and Design (TED), University of Tasmania, Hobart, TAS 7005, Australia; 2 Department of Computer Science, University of Manitoba, Winnipeg, MB R3T 2N2, Canada

**Keywords:** cost model, fog computing, IoT, matching theory, resource allocation

## Abstract

Fog computing aims to support applications requiring low latency and high scalability by using resources at the edge level. In general, fog computing comprises several autonomous mobile or static devices that share their idle resources to run different services. The providers of these devices also need to be compensated based on their device usage. In any fog-based resource-allocation problem, both cost and performance need to be considered for generating an efficient resource-allocation plan. Estimating the cost of using fog devices prior to the resource allocation helps to minimize the cost and maximize the performance of the system. In the fog computing domain, recent research works have proposed various resource-allocation algorithms without considering the compensation to resource providers and the cost estimation of the fog resources. Moreover, the existing cost models in similar paradigms such as in the cloud are not suitable for fog environments as the scaling of different autonomous resources with heterogeneity and variety of offerings is much more complicated. To fill this gap, this study first proposes a micro-level compensation cost model and then proposes a new resource-allocation method based on the cost model, which benefits both providers and users. Experimental results show that the proposed algorithm ensures better resource-allocation performance and lowers application processing costs when compared to the existing best-fit algorithm.

## 1. Introduction

In recent years, Internet of Things (IoT) has become a significant influence for many industries because of various smart features enabled by the advancements in sensor and communication technologies. Recently, Intel announced Xeon D-2100 processor [1] with high computation power with low energy consumption, which is best suited for edge and fog computing environments. According to Gartner [2], the number of IoT devices will reach 20.8 billion by 2020. As the number of devices is rapidly growing and the data generated by these devices are also increasing at an alarming rate, considering the need for processing such data within the time bounds of an application, traditional cloud computing cannot be used due to the limitations such as high latency and limited network bandwidth. Hence, fog computing has evolved as an additional layer to the cloud. Fog computing is envisioned to empower the integration of computation and storage of idle autonomous end devices such as smartphones, tablets, laptops, and other stationary computation devices [3]. Using these idle devices can improve the quality of services needed for executing IoT applications including disaster-related services [4]. In a fog environment, anyone can participate as a provider by providing their computational resources to process time-sensitive applications. For example, recently, the general public contributed their mobile devices for a cancer research project DRUGS (Drug Repositioning Using Grids of Smartphones) [5]. This project aims at using smartphone computing resources to process cancer data. Allocating resources in a vastly distributed heterogeneous device environment is a challenging task due to the variation in available device resources, which can result in variable response and completion times.

Use of resources, cost, and completion time are the parameters that need to be considered during the resource allocation. In the fog computing environment, researchers have only considered the use of resources and response time for allocating the resources without considering the cost [6]. In the literature, there are many cost models and resource-allocation techniques proposed for cloud and other distributed computing paradigms. However, these are not directly applicable in the fog environment as the fog environment has unique characteristics such as high mobility, multiple ownership, and limited resources. Moreover, some works have proposed techniques to allocate the resources in fog by considering energy and latency [7,8]. However, in a realistic fog computing environment, the participating end-user devices (aka fog devices) should be compensated based on the resource usage and the quality of the device to ensure that the devices are reliably available [9]. In such an environment, estimating resource costs before they are allocated and deployed, helps to provide cost-efficient IoT services to the users.

To our best knowledge, no research works have been done that allocate the resources in a fog environment by estimating the cost accurately. On the other hand, cloud resource-allocation techniques and cost models are not suitable for fog because of high heterogeneity and unreliability of fog resources. Since any device that has computation power, can act as a fog device, a fog computing environment is highly heterogeneous compared to the cloud. Moreover, the devices can register and unregister from the network very frequently because of the mobile nature of fog devices. Also, fog devices are not reliable because they are not entirely dedicated to the fog computations; only the idle resources can be used. The owner of the devices offering resources for services in a fog environment should be compensated in some ways for their resource offering. Existing cost models do not consider these fog-related characteristics and user compensation. Hence, a cost model is needed, which can precisely calculate the cost for the users before job submission by considering all these requirements. Moreover, providing an accurate cost estimation model prior to resource allocation is not a trivial task, due to the different resource configurations of fog devices and a large number of highly unreliable and distributed fog resources. As such, this paper tries to fill this gap by proposing a new model that estimates the cost in the fog environment. Based on the proposed cost model, the study further proposes a new resource-allocation algorithm called Fog Stable Matching Resource Allocation (FSMRA) algorithm using stable matching economic theory [10] which benefits both users and providers.

This paper has the following main contributions:

- A micro-level cost model that takes into account user participation in fog computing environments.
- A novel resource-allocation algorithm based on stable matching (FSMRA) is proposed that benefits both users and providers in the fog environment.

The rest of the paper is organized into five sections. Section 2 discusses some of the existing cost models in the literature. Section 3 discusses the problem statement and proposes a solution. Section 4 discusses the fog computing cost model. Section 5 describes the resource-allocation technique with a proposed stable matching algorithm in detail. Section 6 discusses the evaluation of the proposed algorithm. Section 7 concludes the paper.

## 2. Related Work

This section presents the pricing and cost models that are currently available for fog and other related environments such as Cloud and Grid. It then explains why those models are not suitable for the fog environment. The current works related to resource allocation for fog and their limitations are further discussed.

Some works done so far on resource allocation in fog considers the cost as a factor. Ni et al. [11] proposed a resource-allocation strategy in fog where user can choose their required resources automatically. However, the processing costs were not considered in their proposed strategy for credibility evaluation for both users and fog resources. Bellavista et al. [12] proposed a fog framework for resource allocation on Docker Swarm container management that could enable optimal resource allocation through revealed visibility of fog node resources. In [13], the authors proposed a distributed proximal algorithm to solve joint resource allocation with minimizing carbon footprints problem by dividing the globally significant problems into smaller problems. However, their main focus on the paper was to reduce carbon footprints. Similar work was done in [14] where the authors proposed an algorithm for joint resource allocation and minimization problem by offloading tasks to the cloud to exploit the random access networks to meet the requirements of the users. A recent work by Xu et al. [15] proposed dynamic resource allocation in fog along with load balancing. They addressed the cost issues, such as operational cost, migration cost, and hardware cost. They also suggested a load balancing strategy to minimize hardware costs. However, they did not propose any detailed cost model based on the usage of the fog devices.

Previous cost models have not considered the user incentivization. Since fog computing is still evolving and in the early stage of market adoption, according to the best of the authors’ knowledge, there is no specific cost model for fog environments. Some studies have proposed cost models, but they are limited to the Cloud and IoT scenarios. These platforms help users set up and manage the applications and device connections. Some of the pricing models for different IoT-cloud providers available in the market are as follows:

- *IoT Amazon* [16] For IoT, Amazon calculates the cost based on the rules triggered and actions executed. Pricing for IoT Amazon also depends on the number of messages, message size, and connectivity. They charge $0.25 for 5 kb of data. They charge $1.20 for a maximum of one billion messages with each message not exceeding 128 kb. The cost of the connectivity for one million minutes is $0.132.
- *IBM Watson* [17] The pricing model for IBM Watson depends on the number of devices, the number of messages, message size, and percentage of data analytics.
- *AWS Green Grass* [18] The pricing model by AWS Green Grass is based on the number of devices and core. The cost of each device is $0.22 per month.
- *Microsoft Azure IoT Hub* [19] The pricing of Azure IoT depends on the number of messages per day (400000 messages to 6 million per day of size 4 kb for $63).
- *Microsoft Azure Event Hub* [20] The pricing of Azure Event Hub depends on the triggered events ($0.036 per million events), connectivity, and throughput.

There is some related research on cost models and resource allocation in distributed computing paradigms. Rogers and Cliff [21] proposed a resource-allocation technique related to essential resource management of cloud, but, it is very much unclear how the billing should be done. Erdil [22] proposed a resource information sharing approach using proxies. In their approach, proxies deal by sharing resource availability information with the situations where clouds are far away without direct control. The main focus of their study is the sharing of resource information. Kliks et al. [23] presented a cost model for detecting spectrum data. Auxiliary users purchase the data with the goal that they can opportunistically access idle licensed spectrum efficiently. Mei et al. [24] proposed a pricing model for a single seller and multiple buyers based on auctions. In this model, the buyers submit their bidding prices to the auctioning system and the highest bidder is deemed the winner. Park et al. [25] presented a billing model which is mutually verifiable and able to mitigate various types of possible disputes. In their work, they only focused on the transaction reliability for consumption and the purchase of the resources without focusing on the pricing. Sharma et al. [26] proposed a novel cost model, which provides a satisfaction guarantee to both customers and providers in terms of Quality of Service (QoS). Also, the authors used Moore’s law and the compound interest formula to predict the future value of resources based on current values and used the classical Black–Scholes–Merton (BSM) model to calculate the option value. Through their simulation, they investigated the impact of starting an investment, agreement, or term period, the rate of devaluation, the nature of administration, and age of the assets on the asset cost. In recent days, many models for smart data pricing [24,27,28] and techniques have been proposed for Cloud and IoT. In [29], five Sensor Cloud (SC) evaluating Pricing Models (PM) (SCPM1, SCPM2, SCPM3, SCPM4, and SCPM5 ) are proposed. Primarily, they charge an SC client, in view of (1) the rent time frame of the client; (2) the working time required by SC; (3) the SC assets used by the client; (4) the volume of tangible information acquired by the client; (5) the SC way that transmits tactile information from the WSN to the client, separately. Chiang and Zhang [9] initially suggested considering incentivization in cost models, which was later supported by OpenFog consortium [30]. Zheng and Carlee [31] discussed the research problems related to incentivization in their work “Fogonomics: Pricing and Incentivizing Fog Computing”. According to the literature, most of them are concerned with the providers’ perspectives. Some of them are from the perspective of user data quality in IoT. All smart data cost models of cloud do not consider fog resource owners’ participation and compensation to the users, which are crucial in the fog paradigm to enable the real vision of fog computing. Moreover, the characteristics of fog computing differ from the cloud in the heterogeneity of many potentially unreliable devices and lower latency. Therefore, building efficient resource allocation without considering the diversity of the resources and cost estimation model is not cost-effective because failing to do so can lead to improper resource allocation, which in turn leads to the degradation of performance and an increase in the cost.

To the best of our knowledge, there is no research work done yet that considers the fair level allocation of resources with a pricing model which benefits both users and providers.

## 3. Problem Statement and Proposed Solution

The fog computing environment consists of multiple autonomous computational devices from independent users [32]. A user requests for the resources or fog devices to serve the time-sensitive applications. In the fog environments, allocating resources to serve these requests with minimal cost to benefit both users and providers, is a challenging task due to the distributed ownership, decentralized control, and heterogeneity of the fog resources. To address these problems, this study needs to find the answers to the following questions:

1. When the user request is received, how to decide whether the available fog devices can meet the requirements of the user and accept or reject the request?
2. After accepting a user request, how to allocate the resources or fog devices that match the user request’s requirements and simultaneously optimize the cost of an application without affecting the benefits of providers.

To address these questions, firstly a novel cost model is discussed to accurately compute the cost incurred to the user. Based on the users’ resource contribution. Then, the proposed resource-allocation algorithm is discussed.

Each application has minimum requirements of resources while each fog device has a capacity to process the application. From these requirements, the proposed algorithm derives a preference order of resources to process the application. To allocate the resources for processing the application, the proposed algorithm extends stable matching algorithm proposed by Gale and Shapley [10]. The extended algorithm works for an unequal number of fog devices and the users’ requests. The preference of the user list is modelled according to the requirements and the capacity of the fog devices. Using the preference list, the algorithm selects the best matched resources after considering QoS rank and cost. If the resources match more than the requested number of fog devices, it allocates the fog devices which are cost-effective from a user’s perspective. For proportionate distribution of the requests to resource providers, the proposed algorithm chooses the providers who have served the least in the system. The proposed algorithm is evaluated in a simulation environment by varying the number of fog devices and applications.

## 4. Fog Computing Cost Model

Fog computing is a virtualized environment where autonomous end devices form a massively distributed environment to provide various computation-related services by residing between IoT end devices and a conventional cloud environment. In this computing environment, the user services offered to users, depend on peer fog nodes as well as fog providers. For various tasks, the users also participate in the computation process. Hence, this study proposes a new collaborative cost model which benefits both the clients and service providers.

**Figure 1:**
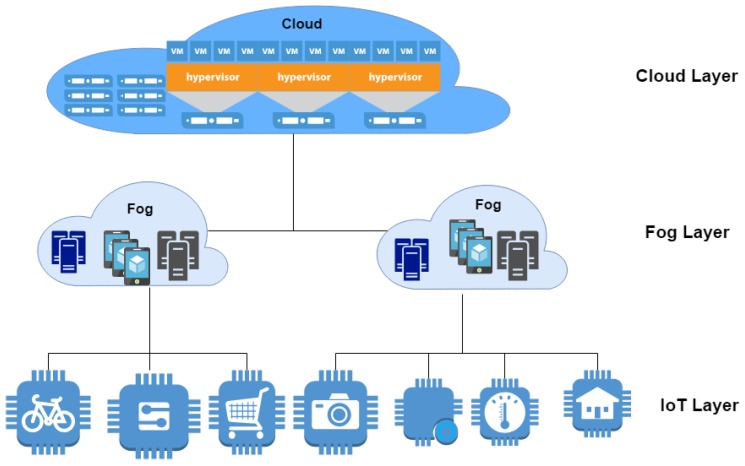
Fog computing.

The general architecture of fog is categorized into three layers [33]: IoT layer, fog layer, and cloud layer, as shown in Figure 1. The IoT layer consists of sensors and the cost of the sensors is calculated based on the number of messages that are transmitted. The fog layer consists of heterogeneous fog devices and the cost of fog devices is divided into storage cost, processing, and network cost with additional factors of scaling and on-demand resources. Cloud layer cost is calculated based on the integration time of fog with cloud (the time required for data exchange between the fog devices and cloud).

The proposed cost model is designed at the micro-level, in all three planes to achieve the most significant benefit for both users and providers. Providers have fog devices which can serve the requests of customers. The computation costs are calculated at the micro-level based on the number of actions triggered and performed, communications of messages and device resources at specific location and time which are used to serve the request while maintaining the required Quality of service (QoS).

The cost of the device or application is given in Equation (1),

(1) $$\begin{array}{c} Cost=\sum_{i=1}^{TD}\left[{Com_{Cost}}_{i}+{Pr_{c}}_{i}+{Net_{c}}_{i}+{Mig_{c}}_{i}+{S_{c}}_{i}+{Pow_{c}}_{i}+{So_{c}}_{i}+{Sen_{c}}_{i}\right]+Op_{c} \end{array}$$

where TD is the total number of devices that participated in the computation. ${Com_{Cost}}_{i}$, ${Pr_{C}}_{i}$ and ${Net_{c}}_{i}$ are the communication cost, processing cost, and cloud-network cost respectively of the device *i*. The cost is calculated for all devices by considering all parameters. Similarly, ${Mig_{c}}_{i}$, ${S_{C}}_{i}$, ${Pow_{c}}_{i}$, ${So_{c}}_{i}$, ${Se_{cost}}_{i}$ and ${Op_{c}}_{i}$ represent migration, storage, power, software, sensors, and operational costs respectively for the device *i*. Each type of cost depends on various parameters and factors. A detailed explanation of each cost is described below. Please note that the unit of time for all the equations is minutes unless explicitly specified.

1. **Communication cost**
  The communication cost depends on the total number of received messages, the size of each message and the message unit cost. Equation (2) shows the formula for calculating communication cost.
  (2) $${Com_{Cost}}_{i}={MR_{total}}_{i}\times \frac{SM_{j}}{x}\times M_{C}$$
  where ${MR_{total}}_{i}$ is the total number of messages received by the device *i*, $SM_{j}$ is the size of the *j*th message in bytes, *x* is the minimum size of the message in bytes defined by the provider and $M_{C}$ is the cost of each message.
2. **Processing cost**
  The processing cost can be calculated in two ways based on user requirements. The user can either request to process the application or specifically request the containers and virtual machines required to process the application.
  The processing cost of application depends on the total number of actions triggered and performed. The processing cost is formulated in Equation (3).
  (3) $$\begin{array}{c} {Pr_{C}}_{i}=\left[NAT_{i}\times QoSF_{i}\times TP_{C}\right]+\left[\left(NAP_{i}+NDT\right)\times QoSF\times AP_{C}\right] \end{array}$$
  where ${Pr_{C}}_{i}$ is total processing cost for *i*, $NAT_{i}$ is the total number of actions triggered, $QoSF_{i}$ is the quality of service factor, $TP_{C}$ is the trigger processing cost, $NAP_{i}$ is the total number of actions performed, NDT is the number of dependent tasks and $AP_{C}$ is the action processing cost.
  The QoS factor depends on the number of actions received, triggered, and executed successfully in terms of meeting the user requirements at a particular location and time. The fog devices behave differently at different times and different places due to the variation in connectivity and the available energy. Hence, QoS of the fog device is calculated based on the previous history of fog devices at a particular time and location in terms of the ratio of the number of successful completion of requests by total number requests triggered. This factor can be calculated by Equation (4).
  (4) $$QoSF_{i}=\frac{\frac{NAT_{i}}{NRR_{i}}+\frac{NAP_{i}}{NAT_{i}}}{2}$$
  where $QoSF_{i}$ is the quality of service factor, $NAT_{i}$ is the total number of actions triggered, $NRR_{i}$ is the total number of requests received and $NAP_{i}$ is the total number of actions performed.
  If the user requests the containers and virtual machines instead of the processing actions-based cost, then the processing cost of the resource is calculated by
  (5) $$\begin{array}{c} {Pr_{C}}_{i}=\left[VCPU_{i}\times QoSF_{i}\times CPU_{U}\right] \end{array}$$
  where $VCPU_{i}$ is the number of VCPU’s and $CPU_{U}$ is CPU usage in hours. The number of VCPU’s can vary from 0.25 to 72.
3. **Cloud-network cost**
  These costs are imposed if coordination with the cloud is necessary. Network costs can be calculated by Equation (6).
  (6) $${Net_{c}}_{i}=CI_{C}\times CIT_{i}$$
  where $CI_{C}$ is the cloud integration pricing and $CIT_{i}$ is the cloud integration time. Cloud integration time is the duration of the time taken by a fog device to send the data to the cloud and receive the data from the cloud after the required computation. This integration time depends on the bandwidth of the network connectivity with the cloud and includes the delay in the process as well.
4. **Migration cost**
  This costs should be paid by the fog contributor who is contributing to fog services if migration takes place due to the fog contributor. However, the participating user should pay migration cost as a penalty if migration is taking place due to the user. Migration cost can be calculated using Equation (7).
  (7) $${Mig_{c}}_{i}=NMT_{i}\times MP_{C}$$
  where $NMT_{i}$ is the total execution time of migration tasks, and $MP_{C}$ is the cost per processing unit.
5. **Storage cost**
  Depend on the volume of data that needs to be stored, the time of storing particular data, and the encryption cost, as shown in Equation (8).
  (8) $${S_{C}}_{i}=TSS_{i}\times EC_{i}\times TS\times STC$$
  where $TSS_{i}$ is the total storage size, EC is the encryption cost per bytes, TS is the storage time in minutes and STC is the storage cost per MB.
6. **Power cost**
  This cost depends on the total number of connected sensors during application processing. The battery cost is calculated using Equation (9).
  (9) $${Pow_{c}}_{i}=\sum_{j=1}^{ND}\left[\left(NSC_{j}\times IT_{j}\times B_{C}\right)\right]$$
  where ND is the total number of fog devices, $NSC_{j}$ is the total number of sensors connected to the fog device, IT is the idle time that is the delay time between sensing and sending in second, and $B_{C}$ is the battery cost.
7. **Software Cost**
  is the commercial production cost which can be calculated by Equation (10).
  (10) $${So_{c}}_{i}=\sum_{i=1}^{z}\frac{\left[{CP_{C}}_{i}\right]}{n_{i}}$$
  where $CP_{C}$ is the commercial product cost per license, *n* is the number of months subscribed and *z* is the number of commercial products.
8. **Sensor cost**
  is calculated by multiplying the number of requests served with the sensor cost per request. Equation (11) shows the sensor cost.
  (11) $${Sen_{c}}_{i}=\sum_{j=1}^{NS}TRS_{j}\times S_{CRj}$$
  where TRS is the total number of requests served by each sensor, NS is the total number of sensors and $S_{CR}$ is the sensor cost per request.
9. **Operational cost**
  depends on the sensor, fog device, and network operation costs, which is formulated as per Equation (12).
  (12) $${Op_{c}}_{i}={SO_{C}}_{i}+{FDO_{C}}_{i}+{NO_{C}}_{i}$$
  where $SO_{C}$ is the sensor operational cost per request, $FDO_{C}$ is the Fog device operational cost per request, and $NO_{C}$ is the network operational cost.

### 4.1. Adjustment Factors

This section discusses the additional factors to consider for the cost in the real-time scenarios such as peak-time cost and scaling cost.

1. **Peak-time cost**
  It is assumed that each device has its demands and it appears at times 1, 2, 3, …, n and ToD is the demand for the device *k* at time *t*. The formula for calculating PTC is given as follows:
  (13) PTC=(1+DU)×Cost
  where PTC is the peak-time cost, which charges during the higher demand of user requests or when the fog devices have met the promised availability but still if the system is requesting the provider to provide for extra time. DU is the demand unit, which represents the rate of extra charges due to the high demand. The formula for calculating DU is given as follows:
  (14) $$DU=\log_{TRA}ToD(k,t)$$
  where TRA is the number of total available resources with the device configuration *k*. The logarithmic function is used in Equation (2) is to make the provider and user participation consistent in all cases.
2. **Scaling cost**
  Scaling cost is needed based on user request, then the user should pay for scaling cost due to the on-demand resources. The scaling of resources can be done in two ways vertical scaling and horizontal scaling. In vertical scaling the resources will be added to the same device by extending the Virtual machine size or container size.
  The vertical scaling cost can be calculated as follows:
  (15) $$SC_{C}=NC_{dev}\times {NA}_{per}\times AP_{C}$$
  where $SC_{C}$ represents the cost of scaling, $NC_{dev}$ is the number of device processors that need to be scaled, $NA_{per}$ represents the number of actions performed and $AP_{C}$ is the cost per action performed. Hence, the total cost with the scaling can be calculated using Equation (17).
  The horizontal scaling cost can be calculated as follows:
  (16) $$SC_{C}=NC_{dev}\times {OD}_{C}$$
  where $SC_{C}$ represents the cost of scaling, $NC_{dev}$ is the number of devices that need to be scaled, ${OD}_{C}$ represents the cost of on-demand resources. Hence, the total cost with the scaling can be calculated using Equation (17).
  (17) $$TS_{C}=Cost+SC_{C}$$

### 4.2. Different Example Scenarios

The proposed cost model helps to calculate the application processing cost or virtual machines (VMs) or container request cost with and without users contributing the resources to serve their requests. There are three different ways of how the users can contribute their resources (as fog device, sensor, and network) to process IoT applications to get incentives. Table 1 shows how the cost is calculated for the provider and user when the user has contributed to different types of resources.

For all these cases the following equations are derived for calculating the cost of fog device (Equation (18)), network (Equation (19)), and sensors (Equation (20)) participation.

(18) $$\begin{array}{c} FD_{C}=\sum_{i=1}^{ND}\left[{Pr_{C}}_{i}+{Mig_{c}}_{i}+{S_{C}}_{i}+{Powcost}_{i}+{So_{c}}_{i}+{FDO_{C}}_{i}\right] \end{array}$$

(19) $$TN_{C}=\sum_{i=1}^{ND}\left[{Com_{cost}}_{i}+{Net_{c}}_{i}\right]+NO_{C}$$

(20) $$S_{C}=\sum_{i=1}^{ND}\left[{Sen_{c}}_{i}\right]+SO_{C}$$

Participating users get rewards based on the resources they contribute. The rest of the total cost is paid to the provider. For example, in Case 1, the user contribution is fog devices and two other resources (e.g., network and sensors). $U_{r}$ denotes the incentives receivable by the user, and $P_{r}$ denotes the incentives receivable by the providers, which are calculated as shown in Equation (21).

(21) $$U_{r}=FD_{C};P_{r}=TN_{C}+S_{C}$$

Similarly, for Case 4 the cost calculation formula will be as per Equation (22).

(22) $$U_{r}=FD_{C}+TN_{C};P_{r}=S_{C}$$

In the same way, this model can calculate how much users and providers are receiving based on their participation in all other cases. For example, if a user request requires the total of fog resources $T_{F}$ and the request is served partially by their own fog nodes $U_{F}$, a peer fog nodes $F_{F}$, and the rest is served by the provider’s resources $P_{F}$, the total cost is calculated as shown in Equation (23).

(23) $$C_{total}=\left(U_{F}/T_{F}\right)+\left(F_{F}/T_{F}\right)+\left(P_{F}/T_{F}\right)$$

If multiple users $NU_{P}$ request services, the cost is divided into equal parts. If a user requests for service and another user also requests for the same service which does not require any extra computations, the price is divided among the users.

(24) $$P_{total}=C_{total}/NU_{P}$$

The incentive that the provider gets is converted by default into the points and these points can be used later for their computations or converted into digital currencies such as BitCoin and Ethers.

### 4.3. Case Study Example

Let us consider an example of a road traffic management application that needs to be deployed in the fog computing environment. Whenever the user requests to find the shortest route from a source to destination, the deployed application collects the data from the sensors installed in vehicles and road-side unit (RSUs) for all possible routes to the requested destination. To process this data, the application selects a single or multiple fog devices based on the sensors from where data needs to be collected and the data transmission frequency of the sensor. In this case, there are no software and migration costs.

The communication cost of the application is calculated based on the data transmitted to and from the fog devices. For example, 100 sensors (50 RSUs and 50 other sensors) are present in a requested route and each sensor transmits the traffic information such as the speed of vehicle and location of size 200 bytes to the fog device FD1 and the provider cost is $0.0001 for a fixed size of 100 bytes. The communication cost based on Equation 2 is $0.02 as shown below.

$${Com_{Cost}}_{i}={MR_{total}}_{i}\times \frac{SM_{j}}{x}\times M_{C}=100\times \frac{200}{100}\times 0.0001=0.02$$

For instance, there are four possible routes for a specified user request. The number of actions triggered for this user request is 100 and the number of actions performed is 4 and the number of dependent actions is 1 to combine all the route and suggest the best possible route. Assume that the cost per action triggered and performed is $0.00015. Based on the history, FD1 has 99% success rate at location L0 and time t1. Hence QoS is 0.99. The processing cost is $0.0155925, as shown below.

$$\begin{array}{c} {Pr_{C}}_{i}=\left[NAT_{i}\times QoSF_{i}\times TP_{C}\right]+\left[\left(NAP_{i}+NDT\right)\times QoSF\times AP_{C}\right]\\ =[100\times 0.99\times 0.00015]+[(4+1)\times 0.99\times 0.00015]=0.155925 \end{array}$$

Assume that the fog device sends the data to the cloud and delete the data once the application is served. FD1 serves the request within 2 s. The storage cost is the duration of time in minutes and the size of the data that is stored in the fog device. 200 bytes message of 100 sensors data is processed for 2 s. If the storage unit cost, MB per minute is 0.000005, the storage cost is $0.00000003 as shown below.

$${S_{C}}_{i}=TSS_{i}\times EC_{i}\times TS\times STC=\left(\frac{200\times 100}{1024\times 1024}\right)\times 1\times \left(\frac{2}{60}\right)\times 0.000005=0.00000003$$

The cloud integration cost is 0.0001 and transfer speed is 100 Mbps then the cloud-network cost is $0.02.

$${Net_{c}}_{i}=CI_{C}\times CIT_{i}=0.0001\times \frac{200\times 100}{100}=0.02$$

Battery cost per request is 0.00005 and 1 millisecond delay the power cost is $0.000005.

$${Pow_{c}}_{i}=\sum_{j=1}^{ND}\left[\left(NSC_{j}\times IT_{j}\times B_{C}\right)\right]=\sum_{j=1}^{1}\left[\left(100\times \frac{1}{1000}\times 0.00005\right)\right]=0.000005$$

The RSUs cost per request is $0.00005 and the other sensors cost per unit is $0.00001 then the total sensor cost is $0.003.

$${Sen_{c}}_{i}=\sum_{j=1}^{NS}TRS_{j}\times S_{CRj}=\left(50\times 0.00005\right)+\left(50\times 0.00001\right)=0.003$$

Operation cost of sensor, fog device and network cost per requests are $0.0000001, $0.0000001 and $0.0000005, respectively. The total operational cost is $0.0000007.

$${Op_{c}}_{i}={SO_{C}}_{i}+{FDO_{C}}_{i}+{NO_{C}}_{i}=0.0000001+0.0000001+0.0000005=0.0000007$$

The total cost of the application is $0.19893073 as shown below.

$$\begin{array}{c} Cost=\sum_{i=1}^{TD}\left[{Com_{Cost}}_{i}+{Pr_{c}}_{i}+{Net_{c}}_{i}+{Mig_{c}}_{i}+{S_{c}}_{i}+{Pow_{c}}_{i}+{So_{c}}_{i}+{Sen_{c}}_{i}\right]+Op_{c}\\ =\sum_{i=1}^{1}\left[0.02+0.155925+0.02+0+0.00000003+0.000005+0+0.003\right]+0.0000007=0.19893073 \end{array}$$

## 5. Proposed Resource Allocation for Fog Computing

Efficient resource allocation is a complex problem in fog environments due to the diversity of the resources. The reason for the diversity is the different resource configurations of various fog devices. The objective of the resource-allocation problem is to find an optimal allocation of the resources to maximize usage and minimize the cost of application processing.

### 5.1. Fog Stable Matching Resource Allocation (FSMRA) Algorithm

This section focusses on a new resource-allocation algorithm called Fog Stable Matching Resource Allocation (FSMRA) Algorithm, which is developed by extending the stable matching algorithm proposed by Gale and Shapley [10]. This algorithm explains the formation of mutually beneficial relationships over time. This model transformed friction matching in economics. The stable matching algorithm cannot be applied directly in fog environments because the stable matching algorithm works only when there is an equal number of resources and requests. This may not be true always in real fog environment. Moreover, the algorithm also does not consider the cost. Hence, this study adapted and extended the stable matching algorithm FSMRA for resource matching and allocation in fog computing environments to serve the maximum number of requests with their requirements by considering the cost of the available resources. The cost model is mutually beneficial to both fog providers and customers and the proposed resource-allocation algorithm is employed in the cost model to serve the application requests with minimal cost.

Based on the “Cobb–Douglas” form of the matching function, Equation (25), calculates the number of fair matching devices that are allocated for the costumers’ application. Based on this information, the request can be accepted or rejected.

(25) $$m_{t}=M\left(u_{t},v_{t}\right)=\mu u_{t}^{a}v_{t}^{b}$$

where μ is the number of sub requests of each application, $u_{t}$ is the number of IoT devices of customers and $v_{t}$ is the number of IoT devices required in peak-time by the fog platform *t*, *a* is the rank of the fog devices to customer. The rank is assigned based on the number of users competing for a particular fog device and *b* is the rank of the fog devices of providers. These matches are based on QoS.

(26) $$n_{t+1}=\mu u_{t}^{a}v_{t}^{b}+\left(1-\delta \right)n_{t}$$

where δ is the fraction of IoT devices that are unavailable or are inefficient, and $n_{t}$ is the total number of IoT devices at time t. The efficiency of fog devices is decided based on the QoS factor specified in Equation (4). If the QoS is less than the user requested QoS, it is treated as inefficient for that request. In fog computing, the devices can join or leave the network at any time, so if the devices are left the network, the nodes are considered as unavailable nodes.

These equations also help to find the number of faulty and inefficient devices and calculate the demand for IoT devices for the next time period.

Existing cost models do not provide any benefits to users for their contributions. However, our proposed cost model provides benefits to the users if they are contributing the resources such as processing, network, and storage. Let us assume that one user acts as both the user of an application and a resource provider, providing both storage and processing resources to execute the application. Then, during billing, the provider deducts the cost of storage and processing from the actual application cost. The proposed cost model considers such scenarios which encourage users to participate in fog processing.

### 5.2. Resource Allocation in Fog

In Fog computing, efficient resource allocation is necessary to provide maximum benefits to users and providers. For example, if an application requests six units of resources but it can assign to a fog device where 10 units of resources are available, in such case user is paying for 6 units but the application request occupies 10 units, and 4 units of resources are idle and hence, wasted. Therefore, it is better to assign application requests to fog devices with the least units of available resources. The matching algorithm is the best solution in such a case. Resource allocation using FSMRA is shown in Algorithm 1. Initially, based on the user requirements, the proposed resource-allocation algorithm gets the available machines list which satisfies the user requirements. During each iteration over the available resource, fog device *fd* checks the preference of user requests *UR*. If *fd* matches the user requirement, it assigns to the particular user request and maps the user request with fog devices if the user preference device has already mapped to the user requests. Then it compares with the device assigned preference with the present user preference. If the present *UR* is more than the assigned *UR* preference, then *fd* is unmapped from the mapping list and maps the *fd* to the current user request. The same process continues for all the fog user requests or until the free available fds list is empty. The final output consists of a list of fds which mapped to the user requests. The overall process chooses for each user request the most preferred fog device list. Moreover, in the next step of the algorithm calculates the estimation cost of fog devices for user requests and selects the least costly devices based on the last served time of the fog devices to get an equal chance of participation in a fog computing environment to process the application.

The proposed algorithm selects a best-suited resource for all user requests based on the request requirements and the resource configuration.

Assume that there are six user requests and six different systems to execute those requests, as shown in Table 2. In the table, AR represents the available resources of fog devices such as Raspberry Pi, Banana Pi, and smartphones. The priority of those requests is given in Table 3. In this table, UR represents the user request. Also assume that the first user request is IO intensive, requesting 2 GB of memory and 300 Kbps network, and the rest of the requests are processing intensive. Processing intensive request requires 2.3, 1.6, 1.5, 1.2 and 1.0 GHz processing power for their operations.

After the employment of the proposed algorithm and best-fit algorithm, machines selected for those six requests, are shown in Table 4. The table also shows the number of successful and failed allocations. According to the scenario mentioned above, all allocations are successfully processed using the proposed algorithm while the best-fit algorithm failed to allocate the resources to serve the application except for the first request. Since the existing algorithm matches only the user requirements with the fog device resources once it finds the best match, it allocates the resources to the application. So, the first request requirements are matched with the first fog device. So, it allocates the resource for the request and the remaining requests are allocated to the nearest matched devices. Hence, it failed to serve all the requests. In the table (Table 4), S is denoting that the requested allocation was satisfied, and NS is denoting the requested allocation was not satisfied. The proposed algorithm always finds the best matches by considering all the parameters of the request with the available resources. In contrast, the best fit only considers the best matching of resources for the allocation.

**Algorithm 1** Resource-allocation algorithm
**Input:**UserRequest<UR>, FreeMachinesList<fd>
**Output:** Pair<UR,fd[]>

freeARs←Getthefreemachineslist

**repeat**
 **while** AvailableResource fd is freeARs **do**  **for all** (UserRequest UR from top of UR’s list) **do**   **if** UR preference is fd **then**    **if** fd is not assigned **then**     Pair( UR,fd[] )←AssignURtofd     pfd=fd    **else if** UR highest preference is fd **then**     the previously assigned pfd     Pair(UR,fd[]) assign UR to fd     unPair(UR,pfd) remove UR to pfd    **else**     unPair(UR,fd) remove UR to fd    **end if**   **end if**  **end for** **end while****until** freeARs list is empty or UR is assigned to fd
**for all** fdfromfd[] **do**
 estimation the cost of the combination of FDs for UR
**end for**
sort the FDs based on the cost and last served timeselect the FDs least cost**return** pair(UR1, fd[])


## 6. Evaluation

This section discusses the evaluation results of the proposed cost model and the resource-allocation algorithm.

### 6.1. Cost Model Evaluation Results

In fog computing, users’ devices are used for computation purposes. However, it is an issue of whether the users agree to participate in the computation process or not. If users receive benefits when their device is being used for fog processing, then the user is more likely to be interested in participating. Likewise, providers also receive benefits in this way. Since the overall cost of processing applications is less if users’ devices are performing some of the computation.

The correctness of the proposed cost model is evaluated numerically and validated by considering a realistic scenario with various participating infrastructures such as sensors and fog devices, network, sensor, and fog devices. The cost is calculated for three use cases: Not Contribution and participation (NCP), Contribution and Not Participation (CNP), and Contribution and Participation (CP). In NCP and CP, the cost is calculated to show the amount that the user needs to pay for the service requested. In CNP, the cost is calculated to show the amount that the owner of the fog device receives for offering the resources.

We adapt the simulation parameters defined in [34] for our realistic scenario, as shown in Table 5. We consider only one scenario as the main aim of the study is the validation of the correctness of the proposed cost model. The user requests to process an application which requires different sensors and different fog devices. The number of participating infrastructures such as fog devices and sensors, is varied during the experiments. Five hundred sensors are generated randomly of random sensor type, with random message size 1–5 kb and the frequency of sending messages in the range of 1–10 messages per second, within the simulation environment. The three types of fog devices medium, small and tiny are created based on the ratio as specified in Table 5 where the total number of fog nodes in the system varies from 75–100. Fog devices process the generated data from sensors to serve an application request based on the maximum capacity (number of actions executed per seconds). Also, we vary the percentage of infrastructure contribution (0–100%) to process the application.

**Figure 2:**
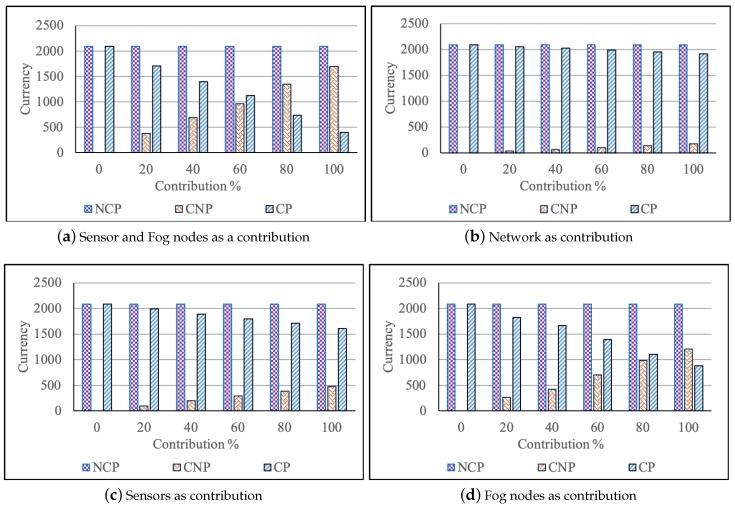
Cost for different cases of infrastructure as a contribution.

Numerical results of our proposed cost model are shown in Figure 2.

Figure 2 represents the costs calculated as per the proposed cost model for the considered scenario with users contributing the sensor and fog nodes as resources. According to this figure, the users must pay higher if they do not have any contribution. If a user has a contribution but does not have any application requests, the user does not need to pay anything but instead receives an incentive for his contribution. In Figure 2a, the user incentives are increasing in CNP with respect to the increase in contribution percentage of fog devices and sensors. In CP case, the user service cost is decreasing with respect to the increase in contribution percentage of fog devices and sensors. However, when the contribution percentage of a user for fog devices and sensors is 100% and the user has some application requests, the user should only pay for the network cost and other operational costs. The users get the highest benefit when they contribute 100% to the application processing and do not have any application request. The same conclusion can be drawn for the other contributions such as network (Figure 2b), the sensor (Figure 2c), and fog device (Figure 2d). The user must pay a little at least as the provider manages the resources and computation process.

By using our cost model, users are getting the benefit of compensation for contributing their fog device for processing the user requests while a traditional cost model does not consider user benefits.

### 6.2. Experimental Evaluation of Proposed Algorithm

This section discusses in detail about the experimental setup and results.

#### 6.2.1. Experimental Setup

The proposed algorithm is validated in the experimental simulation environment by considering various users’ requests and available fog resources. CloudSim [35] is extended so that it can simulate a fog environment. The simulation tool was run on a machine with an Intel Core i7-7600U CPU 2.80 GHz, 2901 Mhz, 2 Core(s), 4 Logical Processor(s) with 16 GB RAM and Windows 10 operating system. For the validation, the best-fit algorithm is used similar to the work done by Abedin et al. [36]. The cost is calculated using currencies (crypto or digital) by increasing the number of device participation and applications. Factors that have been considered for cost calculation are presented in Section 4. Successful completion application percentage is calculated based on the percentage of the number of applications successfully completed by meeting the deadline requirements given by the user compared to the total number of applications submitted. The total number of resources used is computed using the number of fog devices used to serve the bag of applications. The experiments were conducted by considering two cases. In the first case, the number of applications varies from 5 to 50 with random deadlines to complete the applications with at least 10% of the applications are higher deadlines which can only be served in large machine, and the number of fog devices is fixed at 20 which are generated randomly with predefined type tiny, small, medium, and large configurations. In the second case, both the applications and fog resources vary from 5 to 25 with random application deadline requirements and type of the machine.

#### 6.2.2. Results and Findings

**Figure 3:**
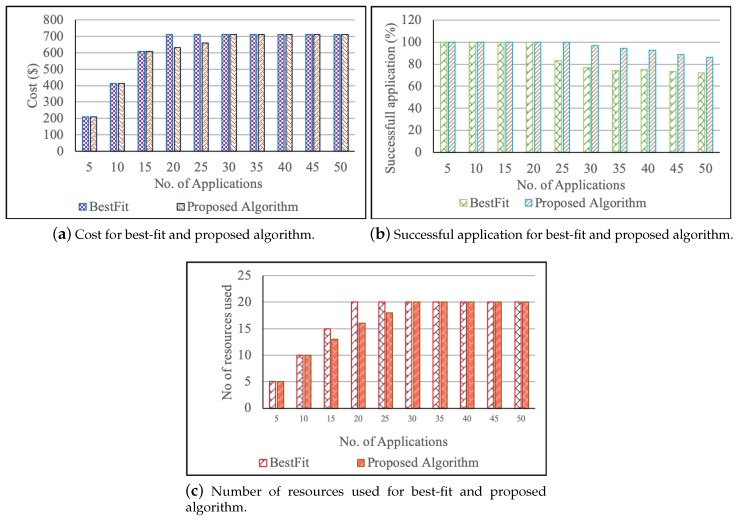
Comparison of cost, resources used, and successful application between the Proposed and BestFit algorithm when the fog devices are fixed.

For the first case, the cost of the proposed and existing algorithm is calculated both by the proposed cost model (PCM). The results show that the proposed algorithm with PCM has less cost compared to the other, as shown in Figure 3a. As the fact that the number of applications is increased, and reaches to the maximum number of fog devices. The proposed algorithm will fit the multiple smaller deadline requirements into the larger machines and medium machines. Once, they select the resources, it finds the cost of the combination of devices. Finally, resource allocator allocates the devices whose cost is minimal. Hence, the cost of the proposed algorithm is better even when the number of tasks is higher than the fog devices.

Figure 3b shows that the number of successful applications is less in best-fit than the proposed solution due to a greater number of applications than the fog resources. The number of resources used to serve the bag of applications is shown in Figure 3c. For all user application, the proposed algorithm always takes fewer resources as the proposed algorithm finds the exact resource for the submitted request by fitting multiple applications in larger machines using the concept of containerization based on preference order. When the number of applications is 25, the best-fit algorithm has fewer successful applications due to the fact that the resources are allocated to the application without considering the other requests in the queue and all the other parameters. For example: Assume two resources are available with the configurations of (2.3 GHz processor, 300 Kbps network, and 2 GB RAM) and (1.6 GHz processor, 300 Kbps network, and 2 GB RAM). If the first request is IO intensive ( requiring 300 Kbps network and 2 GB RAM) and the second request is compute intensive (requiring 2.3 GHz and 2 GB RAM), the existing best-fit algorithm allocates the first resource for the first request as it best fits the request. The second request does not have any fitting resources and hence fails due to resource unavailability. However, the proposed algorithm overcomes this problem by evaluating the preference of resources by considering all the parameters of requests and hence, allocates the second request to the first configuration and first request to the second configuration. The proposed algorithm successfully serves all the incoming requests by finding the perfect matches, which is not existent in similar best-fit algorithms.

For a fixed number of fog nodes (20), when the number of application is 30 or more, the application requests with smaller deadlines fit into the unused resources available within the system, which increases the resource use. On the other hand, the best-fit algorithm takes a longer time for the requests that do not correctly fit the resources. In fog, meeting the user requirements is essential as the fog is evolved for the time-sensitive applications.

**Figure 4:**
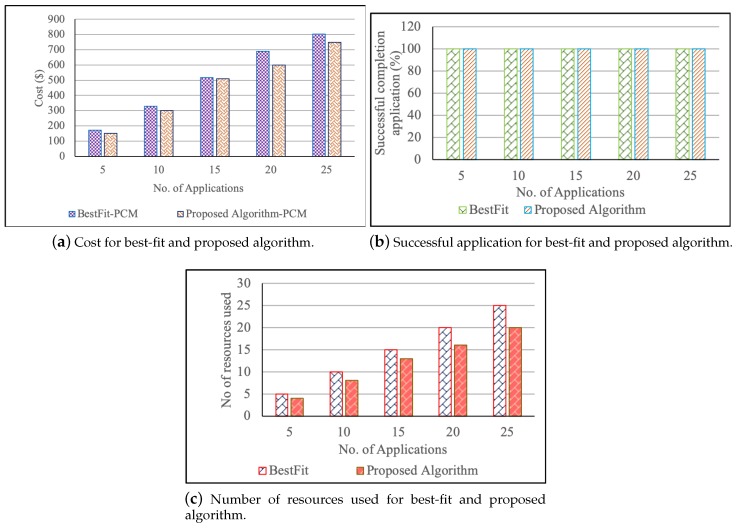
Comparison of cost, resources used and successful application between the Proposed and BestFit algorithm when the fog devices are equal to number of applications.

In the second case, the total cost to serve the bag of applications of both proposed and existing algorithms are calculated with PCM. The results show that the user needs to pay more in the existing resource-allocation algorithm. Moreover, the cost is less in the proposed algorithm with PCM in all the cases, as shown in Figure 4a. As the number of applications and devices are increasing, the proposed algorithm will adjust the containers of larger fog resources to use the resources fully and selects the least cost devices to serve the applications. Figure 4b presents the number of successful completion of applications. The rate of a successful application is 100% in both cases because they have enough resources which can meet the requirements of applications. Figure 4c represents the number of resources to serve the bag of applications. In the proposed algorithm, the resources used are fewer compared to the proposed system. Hence, it indicates that the employment of the proposed algorithm always incurred less cost and gives the equal opportunity to the devices to serve the user requests, which is beneficial for users as well as for the providers.

## 7. Conclusions and Future Work

Fog computing has evolved to process the user application services either in the peer fog nodes or in fog service providers’ devices or both. This study proposed a model for micro-level cost estimation and FSMRA algorithm based on the proposed cost model for resource allocation that benefits both the customers and the providers. The study considered the user incentivization to the users who offer their resources in the proposed cost model, which is one of the most crucial aspects of fog computing paradigm. The proposed resource-allocation algorithm, based on the cost model, offers fair and equal opportunities to the resource providers to serve the application requests in an efficient manner(minimal cost), thereby benefiting the users and the providers. Experimental and simulation results showed that the proposed resource-allocation algorithm can select minimal, suitable resources to meet user requirements with less cost compared to the best-fit algorithm.

In a fog computing environment, any user can participate by providing their devices such as mobile devices, laptops, iPads, IoX-included switches and routers for processing the IoT applications such as mhealth, visual security (public safety), e-learning environments, and smart cities (traffic controlling). As a new kind of business model has evolved in the environment, the way of economic interaction between the users and the service/resource providers must change. For effective implementation of cost models, the usage of the resources and the benefits to the service/resource providers must be quantified accurately, which can be a challenging task. Moreover, when general people as resource provider allow access into their devices, security and privacy issues may arise.

In the proposed resource-allocation algorithm, if a new incoming request is unable to fit in with unallocated available resources, the system must recalculate the mapping of all the applications to the resources. This can increase the time required to calculate the resource-allocation plan within the system.

As future works, a system, based on the proposed cost model, will be designed to automate the payments between the distributed service providers and users by using blockchain. Dynamic user requirements, fault tolerance and scalability for building efficient fog resource management system can be considered for future extensions.

## Figures and Tables

**Table 1:** Different cases of users and providers contribution.

Cases	User’s Contribution	User Request	Cost
1	Fog devices, Sensors	N	$Cost\left(CutomerPay\right)=\sum_{i=1}^{ND}\left[{Pr_{c}}_{i}+{Mig_{c}}_{i}+{S_{C}}_{i}+{Pow_{c}}_{i}\right]+{So_{c}}_{i}+{Sen_{c}}_{i}]+{Op_{c}}_{i}-{NO_{C}}_{i}$
2	Fog devices, Sensors	N	$Cost\left(ProviderPay\right)=\sum_{i=1}^{ND}\left[{Com_{c}}_{i}+{Net_{c}}_{i}\right]+{NO_{C}}_{i}$
3	Network	N	$Cost\left(CustomerPay\right)=\sum_{i=1}^{ND}\left[{Com_{c}}_{i}+{Net_{c}}_{i}\right]+{NO_{C}}_{i}$
4	Network	Y	$Cost\left(ProviderPay\right)=\sum_{i=1}^{ND}\left[{Pr_{c}}_{i}+{Mig_{c}}_{i}+{S_{C}}_{i}+{Pow_{c}}_{i}\right]+{So_{c}}_{i}+{Sen_{c}}_{i}]+{Op_{c}}_{i}-{NO_{C}}_{i}$
5	Sensors	N	$Cost\left(CustomerPay\right)=\sum_{i=1}^{ND}\left[{Sen_{c}}_{i}\right]+{SO_{C}}_{i}$
6	Sensors	Y	$Cost\left(ProviderPay\right)=\sum_{i=1}^{ND}\left[{Pr_{c}}_{i}+{Mig_{c}}_{i}+{S_{C}}_{i}+{Pow_{c}}_{i}\right]+{So_{c}}_{i}+{Op_{c}}_{i}-{SO_{C}}_{i}]$
7	Fog devices	N	$Cost\left(CutomerPay\right)=\sum_{i=1}^{ND}\left[{Pr_{c}}_{i}+{Mig_{c}}_{i}+{S_{C}}_{i}+{Pow_{c}}_{i}\right]+{So_{c}}_{i}+{FDO_{C}}_{i}]$
8	Fog devices	Y	$Cost\left(ProviderPay\right)=\sum_{i=1}^{ND}\left[{Com_{c}}_{i}+{Net_{c}}_{i}+{Sen_{c}}_{i}+{Op_{c}}_{i}-{FDO_{C}}_{i}\right]$
9	None	N	$Cost\left(ProviderPay\right)=\sum_{i=1}^{TD}\left[{Com_{Cost}}_{i}+{Pr_{C}}_{i}+{Net_{c}}_{i}+{Mig_{c}}_{i}+{S_{C}}_{i}+{Pow_{c}}_{i}+{So_{c}}_{i}+{Se_{cost}}_{i}\right]+{Op_{c}}_{i}]$

**Table 2:** Available resources of machines.

Resource ID	Processor (GHz)	Network (Kbps)	RAM (GB)	Cost
AR1	2.3	300	2	0.008
AR2	1.6	300	2	0.006
AR3	1.5	1000	1	0.004
AR4	1.2	150	1	0.002
AR5	1.0	300	1	0.001
AR6	0.9	1000	2	0.007

**Table 3:** Resource priority of the users.

User Request No	Pr1	Pr2	PR3	PR4	PR5	PR6
UR1	AR6	AR2	AR1	AR3	AR4	AR5
UR2	AR1	AR2	AR3	AR4	AR5	AR6
UR3	AR2	AR1	AR3	AR4	AR5	AR6
UR4	AR3	AR2	AR1	AR4	AR5	AR6
UR5	AR4	AR3	AR2	AR1	AR5	AR6
UR6	AR5	AR4	AR3	AR2	AR1	AR6

**Table 4:** Selected machined after applying the proposed algorithm and best-fit algorithms.

User Request No	Proposed Algorithm		Best-Fit	
	System	Status	System	Status
UR1	AR6	S	AR1	S
UR2	AR1	S	AR2	NS
UR3	AR2	S	AR3	NS
UR4	AR3	S	AR4	NS
UR5	AR4	S	AR5	NS
UR6	AR5	S	AR6	NS

**Table 5:** Evaluation parameters.

Parameter	Description	Value	Type
N	Number of Source nodes	500	temperature and proximity
M	Number of Fog nodes	75–100	medium, small and tiny
R	Ratio of Fog nodes	5–10%, 30–35%,remaining%	medium, small and tiny
lh	Hop delay	10 [ms/hop]
C	Maximum capacity of one Fog node	5, 10, 15, [actions executed]	tiny, small, medium
λ	Producing work rate	10–100 [actions executed/s]	temperature and proximity
QoSF	QoS Factor	90–100%	Fog nodes
NW	Network speed	300–1000 Kbps	Fog nodes

## Abbreviations

The following abbreviations are used in this manuscript:

*ND*	Total number of devices	*TSS*	Total storage size
$ST_{C}$	Storage time cost	*NDT*	Number of dependent tasks
*NAT*	Total number of actions triggered	*M*	Total number of messages received
*NRR*	Total number of requests received	*NSC*	Total number of sensors connected
*NAP*	Total number of actions performed	*IT*	Idle time
$M_{C}$	Messaging cost	$B_{C}$	Battery cost
$E_{C}$	Encryption cost	$CP_{C}$	Commercial product cost
*QoSF*	Quality of service factor	*TRS*	Total requests served
*TP_C_*	Trigger processing cost	$S_{CR}$	Sensor cost per request
*AP_C_*	Action processing cost	*DU*	Demand unit
*CI_C_*	Cloud integration cost	*ToD*	Total demand
*CIT*	Cloud integration time	*TRA*	Total resource availability
$Net_{c}$	Network cost	$SO_{C}$	Sensor operational cost
*Cost*	Overall Cost	$NO_{C}$	Network operational cost
*MP_C_*	Migration processing cost	$TS_{C}$	Total cost with scaling
$FDO_{C}$	Fog device operational cost	*NM*	Total number of tasks migration
$NC_{dev}$	Number of the devices need to be scaled	$S_{C}$	Storage cost
$TN_{C}$	Total network cost	$Pow_{c}$	Power cost
$So_{c}$	Software Cost	$Sen_{c}$	Sensor cost
$Op_{c}$	Operational cost	$FD_{C}$	Fog device cost
$S_{C}$	Sensor cost	$U_{r}$	User receiving
$P_{r}$	Provider receiving	$AT_{C}$	Cost per action triggered
$Mig_{c}$	Migration cost	$NA_{per}$	number of actions performed
$Pr_{C}$	Processing cost	$SC_{C}$	Cost of scaling
TS	Storage time	$OD_{C}$	On-demand Cost
